# Immunotherapy for pancreatic cancer: Barriers and breakthroughs

**DOI:** 10.1002/ags3.12176

**Published:** 2018-06-22

**Authors:** Robert J. Torphy, Yuwen Zhu, Richard D. Schulick

**Affiliations:** ^1^ Department of Surgery University of Colorado Aurora Colorado USA

**Keywords:** cancer vaccine, immune checkpoint, immunotherapy, pancreatic cancer, tumor microenvironment

## Abstract

Immunotherapy is a rapidly growing field and represents a paradigm shift in the treatment of malignancies as it offers a new therapeutic approach beyond surgery, conventional chemotherapy, and radiation treatment. Targeting immune checkpoints, such as cytotoxic T‐lymphocyte‐associated antigen 4 and programmed death 1/programmed death ligand 1 has had immense clinical success resulting in sustained treatment response for a subset of patients with certain malignancies such as melanoma, non‐small‐cell lung cancer, urothelial carcinoma, squamous cell carcinoma of the head and neck, renal cell cancer, hepatocellular cancer, and metastatic colorectal cancer. Importantly, there has been limited success in the use of immunotherapy in the treatment of pancreatic cancer. Investigation into the complex tumor microenvironment of pancreatic cancer that is composed of immune cells, stromal cells, and extracellular matrix proteins has begun to shed light on important attributes of this microenvironment that act as barriers to the effective use of immunotherapy. In this review, we will discuss the progress that has been made in treating pancreatic cancer with immunotherapy, the barriers that have limited treatment success, and breakthroughs with combination treatments that hold promise for the future.

## 1 INTRODUCTION

Pancreatic cancer is projected to be the third leading cause of cancer‐related death in the USA in 2018.^1^ Significant improvements in the treatment of pancreatic cancer have been made over the past two decades, with a median survival of 28.0 months now reported for patients with resectable disease who receive adjuvant gemcitabine plus capecitabine.^2^ Although outcomes have improved dramatically for patients with resectable disease who receive adjuvant chemotherapy, there has been very limited advancement in treating metastatic pancreatic cancer since gemcitabine was shown to be superior to 5‐fluorouracil in 1997.^3^ Since that time, the addition of nab‐paclitaxel to gemcitabine therapy and FOLFIRINOX have been shown to be alternative treatment strategies, but survival benefits with these treatments are modest and overall survival remains poor.^4^

Although the treatment of pancreatic cancer has evolved slowly over the past two decades, treatment of other malignancies has experienced immense progress through the breakthrough of cancer immunotherapy. However, the success of immunotherapy has not translated to the treatment of pancreatic cancer which has been shown to be unresponsive to anti‐programmed death 1 (anti‐PD‐1) and anti‐cytotoxic T‐lymphocyte‐associated antigen 4 (anti‐CTLA‐4).^5^, ^6^

The ineffectiveness of immunotherapy in pancreatic cancer may be explained by these tumors being non‐immunogenic.^7^ One explanation for the non‐immunogenic nature of these tumors is their poor antigenicity. A key initial step of launching effective anti‐tumor immunity is tumor antigenicity; T cells must be able to recognize malignant cells as foreign to elicit their destruction. The antigenicity of tumors can be, in part, inferred by their mutational landscape. Solid tumors such as pancreatic cancer, colon cancer, and breast cancer contain an average of 33 to 66 somatic mutations that result in altered protein production and, in turn, could lead to the expression of “foreign” antigens. Conversely, melanoma and lung cancer have approximately twice as many somatic mutations making them relatively more antigenic.^8^ Despite the decreased antigenicity of pancreatic cancer when compared to melanoma and lung cancer, multiple studies have indicated that the human immune system can develop an immune response to pancreatic cancer and generate functional anti‐tumor T cells.^9^, ^10^ This suggests that other mechanisms are likely contributing to the non‐immunogenic nature of pancreatic cancer.

It is well known that the interaction of the immune system and cancer cells is a dynamic process that can eliminate antigenic cancer cells, but can also evolve to a phase where cancers escape from immune surveillance.^11^ The process of immune escape is multifactorial but involves reduced immune recognition of cancer cells and the development of an immunosuppressive microenvironment. There is an increasing body of knowledge that suggests the tumor microenvironment of pancreatic cancer is very effective in promoting immune escape, rendering the immune system unable to mount an effective anti‐tumor response. Understanding the role of the tumor microenvironment in facilitating immune escape in pancreatic cancer holds great potential for improving the success of immunotherapy for pancreatic cancer in the future.

In this review we will briefly describe the limited success that has been seen with immune checkpoint blockade and pancreatic cancer vaccines and explore the unique tumor microenvironment of pancreatic cancer that acts to limit the effectiveness of immunotherapy. Last, we will highlight how the tumor microenvironment can be targeted in combination with immunotherapy to unleash the potential of immunotherapy as an effective treatment modality in pancreatic cancer.

## 2 LIMITED SUCCESS OF TRADITIONAL IMMUNOTHERAPY

### 2.1 Immune checkpoint blockade

Cytotoxic T‐lymphocyte‐associated antigen 4 is a coinhibitory receptor expressed on activated CD4^+^ and CD8^+^ T cells and competes with CD28 for ligands B7‐1 and B7‐2. Blockade of CTLA‐4 can induce anti‐tumor immunity.^12^ Ipilimumab is a fully human monoclonal antibody (mAb) that improves overall survival in patients with metastatic melanoma and elicits a long‐term survival benefit in a subset of patients.^13^ Ipilimumab became the first immune checkpoint‐targeted therapy to receive approval for clinical use in the USA and Europe in 2011. Ipilimumab treatment was evaluated in a phase II study in patients with advanced pancreatic cancer in 27 patients and showed a delayed response in one patient only, indicating that single‐agent ipilimumab was not an effective therapy in advanced pancreatic cancer.^5^

Programmed death 1 was the second coinhibitory receptor to come to the forefront of cancer immunotherapy. PD‐1 is expressed by effector T cells, regulatory T (Treg) cells, B cells and natural killer (NK) cells and binds to the ligands programmed death ligand 1 (PD‐L1; B7‐H1) and programmed death ligand 2 (PD‐L2; B7‐DC). Upon binding to these ligands, PD‐1 acts to inhibit T cells.^14^ PD‐L1 is expressed both by cancer cells and tumor‐infiltrating lymphocytes and upregulation of PD‐L1 on tumor cells is an adaptive mechanism to facilitate immune evasion. PD‐1 mAb therapy with pembrolizumab and nivolumab have had great clinical success for a variety of solid tumors and have received Food and Drug Administration (FDA) approval for the treatment of multiple solid tumors, including melanoma, non‐small‐cell lung cancer, urothelial carcinoma, and renal cell cancer.^15^, ^16^ Atezolizumab is an anti‐PD‐L1 monoclonal antibody that can also disrupt the interaction between PD‐1 and its ligands,^17^ and has been approved for the treatment of non‐small‐cell lung cancer and urothelial carcinoma.^18^

Multiple human studies have indicated that high PD‐L1 expression in pancreatic cancer tumors is associated with worse outcomes suggesting that targeting the PD‐1/PD‐L1 interaction may have therapeutic benefit in these patients.^19^, ^20^, ^21^ An early preclinical study in a mouse tumor transplant model showed that PD‐1 or PD‐L1 blockade had an anti‐tumor effect which was enhanced when given together with gemcitabine.^19^ Unfortunately, these preclinical findings have not translated to clinical success. In a phase I trial of anti‐PD‐L1 therapy, no patients with pancreatic cancer showed a clinical response.^6^

### 2.2 Vaccine therapy

Cancer vaccines are designed to augment antigen presentation and activate antigen‐specific effector and memory T cells.^22^ When vaccines containing target tumor antigens are given, host antigen‐presenting cells (APC) are tasked with taking up these antigens for presentation to effector T cells which are then primed to kill tumor cells expressing these antigens. The desired result is the development of anti‐tumor immunity. Several pancreatic cancer antigens have been identified that are shared by the majority of pancreatic tumors and include carcinoembryonic antigen (CEA), mucin‐1 (MUC‐1), and the product of mutated KRAS.^23^ All these antigens have the potential to be used as vaccines for pancreatic cancer. In addition, allogenic tumor cell vaccination, using tumor cell lines, can induce effective and tumor‐specific immune responses in mouse tumor models.^24^

GVAX is a whole‐cell vaccine in which pancreatic cancer cells are engineered to express the proinflammatory cytokine granulocyte monocyte‐colony stimulating factor (GM‐CSF) to further stimulate APC antigen uptake and T‐cell priming.^25^ A phase I study evaluated the safety and efficacy of GVAX as an adjuvant therapy given in series with chemoradiation therapy in patients with resected pancreatic cancer. This phase I study showed that vaccination with GVAX was safe. Data from this trial also provided evidence that GVAX was effective in promoting the development of anti‐tumor immunity, as vaccinated patients developed a dose‐dependent delayed‐type hypersensitive reaction against autologous tumor cells.^23^ This immunotherapy approach was further evaluated in a phase II study of 60 patients and their outcomes were compared to historical controls. Patients who received adjuvant vaccine therapy in conjunction with chemoradiation did not show improved 1‐year or overall survival compared to historical controls treated with adjuvant chemoradiation therapy alone. However, a subgroup of patients with prolonged disease‐free survival had increased tumor antigen‐specific CD8^+^ T cells after their final vaccination suggesting this treatment approach may be effective for certain patients.^26^

An alternative to whole‐cell vaccines are peptide‐based vaccines, an example of which is the peptide product of mutated *KRAS*. The *KRAS* gene is mutated in over 90% of pancreatic adenocarcinomas, making it a vaccine target that would be broadly applicable for the treatment of pancreatic cancer.^27^ A phase I/II trial of a mutated RAS peptide vaccine in conjunction with GM‐CSF in patients with resected or advanced pancreatic cancer showed that this treatment strategy elicited an anti‐RAS immune response in 58% of patients. Further, median survival in those who developed an anti‐RAS immune response was more than double those who did not develop a response.^28^ A recent phase II study of 30 patients from Japan also indicated safety and efficacy of an adjuvant multipeptide vaccine and chemotherapy regimen following surgical resection. The multipeptide vaccine, OCV‐C01, contained peptides derived from vascular endothelial growth factor receptor (VEGFR)1, VEGFR2 and a kinesin‐family protein (KIF20A). This regimen was well tolerated and, importantly, 58.6% of patients developed a cytotoxic lymphocyte response against KIF20A. In the per‐protocol analysis, patients who developed a KIF20A‐specific cytotoxic lymphocyte response had a significantly improved disease‐free survival.^29^ These promising results indicate that vaccine therapy can be a useful immunotherapy in the treatment of pancreatic cancer, but more work is needed to identify key biomarkers that predict response, and to optimize combinatory therapies to increase the effectiveness for all patients.

### 2.3 Vaccine and immune checkpoint blockade in combination

A phase I study compared ipilimumab and ipilimumab in combination with GVAX in locally advanced and metastatic pancreatic cancer. This study included 30 patients, randomized 1:1, and showed that combination therapy was safe with evidence favoring increased efficacy with combination therapy. Two patients in the ipilimumab‐alone arm showed stable disease at 7 and 22 weeks, whereas three patients in the combination arm had prolonged disease stabilization at 31, 71, and 81 weeks. Patients from both treatment arms who showed prolonged overall survival had higher levels of mesothelin‐specific CD8^+^ T cells in peripheral blood samples after treatment indicating an improved anti‐cancer T‐cell response.^30^

Preclinical studies in melanoma indicated that GM‐CSF‐producing vaccines in combination with immune checkpoint blockade was able to effectively treat a non‐immunogenic melanoma cancer cell line that did not respond to immune checkpoint blockade therapy alone.^31^ A similar treatment modality was tested in preclinical models of pancreatic cancer using GVAX in combination with anti‐PD‐1 therapy. Combination therapy was found to significantly increase median overall survival compared to PD‐1 monotherapy with a trend towards improved overall survival. Tumor infiltrating lymphocytes (TIL) collected from the tumor microenvironment of mice that received combination therapy had an increase in the percentage of interferon (IFN)‐gamma‐producing CD8^+^ T cells compared with single‐agent therapy, indicating a synergistic effect on anti‐tumor immunity.^32^ A possible mechanism for this synergy is that vaccination induces some anti‐tumor immunity that is overcome by upregulation of PD‐L1 on tumors. Patients who received the GVAX vaccine 2 weeks prior to surgical resection had an increased frequency of tumors positive for PD‐L1 (12.5% of resected tumors in the unvaccinated group vs 25% of resected tumors in the GVAX group), suggesting this as a likely mechanism of immune resistance.^32^ Building on these results, GVAX is currently being studied in a clinical trial with or without nivolumab for patients with resectable pancreatic cancer at Johns Hopkins University (NCT02451982; clinicaltrials.gov).

## 3 TUMOR MICROENVIRONMENT AS A BARRIER TO IMMUNOTHERAPY

**Figure 1:**
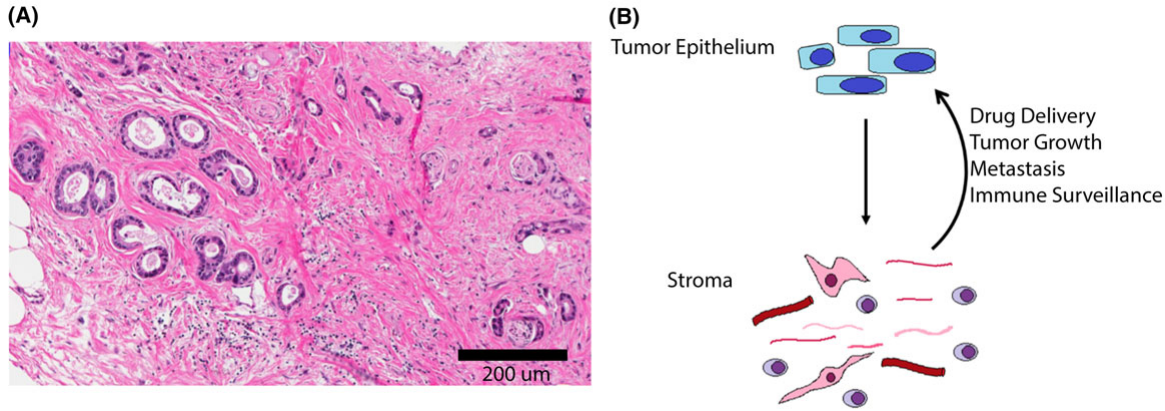
Abundant desmoplastic stroma of pancreatic cancer. A, Hematoxylin and eosin‐stained primary pancreatic ductal adenocarcinoma (10×), showing abundant desmoplastic stroma surrounding tumor epithelial cells. B, Interaction of tumor epithelial cells and stromal components can impede drug delivery, enhance or impair tumor growth and metastasis, and alter immune surveillance of tumors

Pancreatic cancer tumors are composed of malignant cancer cells surrounded by an abundant desmoplastic stroma (Figure 1A). This desmoplastic stroma forms the tumor microenvironment of pancreatic cancer and is composed of fibroblasts, pancreatic stellate cells, immune cells, blood vessels, and extracellular matrix proteins.^33^ This abundant stroma has been implicated as a physical barrier in pancreatic cancer that plays a role in preventing effective delivery of standard chemotherapies to tumors.^34^ Depleting stroma in preclinical mouse models of pancreatic cancer through inhibiting the Hedgehog cellular signaling pathway was shown to improve delivery of gemcitabine to tumors and resulted in improved survival.^35^ However, these results did not translate to clinical success. A phase II clinical trial of gemcitabine with vismodegib, a Hedgehog antagonist used to deplete stroma, showed no survival benefit in pancreatic cancer patients.^36^ Subsequent studies have reported stroma depletion may actually enhance tumor growth underscoring the complex role that stroma plays in tumor biology.^37^ Despite the conflicting results of tumor response to stroma‐depleting therapies, the tumor microenvironment plays a significant role in tumor biology and in modulating the immune recognition of pancreatic cancer (Figure 1B).

### 3.1 Immunosuppressive immune cells in the tumor microenvironment

Genetically modified mouse models that develop spontaneous pancreatic tumors are an important model for studying the effects of the tumor microenvironment on response to immunotherapy. One such model is the KPC mouse, which is genetically engineered to express mutant *Kras* and mutant *p53* in the pancreas.^38^ This model develops pre‐invasive lesions (pancreatic intraepithelial neoplasia) which progress to invasive and metastatic disease, recapitulating the progression of human disease. These spontaneous tumors also have an abundant desmoplastic stroma reaction like that found in human tumors.^39^ Evaluation of the immune response in pre‐invasive and invasive lesions from these mouse models showed that leukocyte invasion is present throughout the progression of disease.^40^, ^41^, ^42^ However, this leukocyte invasion was dominated by immunosuppressive cell types (tumor‐associated macrophages, myeloid‐derived suppressor cells [MDSC], and Treg cells) and lacked effector T cells.^40^ These findings are significant as they indicate a suppressed immune response from the earliest development of pre‐invasive lesions as opposed to an activated immune response that is overcome by the tumor developing resistance mechanisms.

The prevalence of these immunosuppressive cell types and their prognostic significance has also been shown in humans. Patients with pancreatic cancer have elevated levels of Treg and MDSC in peripheral blood samples when compared to healthy controls and elevated levels of these immunosuppressive cells are associated with a poor prognosis.^43^, ^44^ In the tumor microenvironment of the pancreas, Treg, MDSC, and M2‐polarized tumor‐associated macrophages are also increased and are associated with a poor prognosis.^44^, ^45^, ^46^, ^47^

### 3.2 Immunosuppressive function of carcinoma‐associated fibroblasts

Along with the immunosuppressive immune cells found in the tumor microenvironment of pancreatic cancer, these tumors contain an abundant stroma of mesenchymal origin rich in fibroblasts. Carcinoma‐associated fibroblasts of epithelial malignancies have been shown to express fibroblast activation protein‐α (FAP).^34^ In pancreatic cancer, high FAP expression in tumors correlates with worse prognosis.^34^, ^48^ Kraman et al^49^ further investigated the potential of FAP to have immunosuppressive properties in the tumor microenvironment using a transgenic mouse model depleted of FAP‐positive cells. Mice that were depleted of FAP+ cells and harboring subcutaneous tumors showed tumor regression after treatment with a vaccine‐based immunotherapy. In contrast, wild‐type mice did not show tumor regression, suggesting depletion of FAP+ cells enabled the efficacy of this vaccine‐based immunotherapy. In a separate study using KPC mice, depletion of FAP+ stromal cells was synergistic with anti‐CTLA‐4 or anti‐PD‐L1 therapy, further demonstrating the role of stroma in suppressing anti‐tumor immunity.^50^

## 4 BREAKTHROUGHS WITH COMBINATION THERAPIES

The tumor microenvironment of pancreatic cancer presents a therapeutic barrier in treating pancreatic cancer with traditional immunotherapies. However, treating pancreatic cancer with combination approaches that reprogram the tumor microenvironment and ultimately unleash the potential benefits of immunotherapy are now being pursued with promising preliminary results.

### 4.1 Colony‐stimulating factor 1 receptor blockade to reprogram the immunosuppressive microenvironment

In the tumor microenvironment, colony‐stimulating factor 1 receptor (CSF1R) is expressed on tumor associated macrophages and MDSC which can play critical roles suppressing the cytotoxic immune response. Investigation of the expression of CSF1R and its ligand, CSF1, indicating that CSF1 is frequently expressed by pancreatic cancer cells whereas CSF1R expression is limited to cells in the tumor microenvironment, suggesting a tumor cell/tumor microenvironment interaction. Blockade of CSF1R has been shown to improve chemotherapy‐induced antitumor immunity in mouse cancer models leading to the hypothesis that targeting the CSF1R/CSF1 interaction in combination with immune checkpoint blockade could have a synergistic response.^51^, ^52^

In mouse pancreatic cancer tumor transplant models, treatment with CSF1R blockade resulted in a reprogramming of the immunosuppressive tumor microenvironment. Following treatment with CSF1R blockade by a CSF1R tyrosine kinase inhibitor tumor, infiltrating macrophages and MDSC were depleted. Treatment of tumor‐bearing mice with CSF1R blockade did result in enhanced effector T‐cell tumor infiltration and reduced tumor growth. However, following treatment, tumor cells had increased expression of PD‐L1 and effector T cells had increased expression of CTLA4, suggesting immune checkpoint blockade in conjunction with CSF1R blockade may have a synergistic effect. Combination therapy with gemcitabine, CSF1R blockade and either anti‐CTLA4 or anti‐PD1 therapy resulted in a synergistic response that was further enhanced with co‐blockade of both PD‐1 and CTLA‐4 with complete tumor regression in 30% of animals and an average tumor regression of 85%.^52^

IMC‐CS4 is a CSF1R antibody currently in phase I clinical trial in conjunction with GVAX and anti‐PD1 therapy for borderline resectable pancreatic cancer. PLX‐3397, commercially named Pexidartinib (Plexxikon Inc., Berkeley, CA, USA), is another anti‐CSF1R agent that is currently in phase I clinical trial in conjunction with anti‐PD‐L1 for patients with advanced pancreatic and colorectal cancer (Table 1).

**Table 1:** Active clinical trials of novel or combination immunotherapy approaches for the treatment of pancreatic cancer

Treatment	Phase	Patient population	ClinicalTrials.gov identifier
GVAX CSF1R antibody (IMC‐CS4) Anti‐PD1 (Pembrolizumab) Cyclophosphamide	1	Borderline resectable pancreatic cancer	NCT03153410
Anti‐PD‐L1 (Durvalumab) CSF1R inhibitor (Pexidartinib)	1	Advanced pancreatic and colorectal cancer	NCT02777710
CXCR4 antagonist (Plerixafor)	1	Advanced pancreatic cancer	NCT03277209
CD40 agonist (R07009789) Nab‐Paclitaxel Gemcitabine	1	Resectable pancreatic cancer	NCT02588443

CSF1R, colony‐stimulating factor 1 receptor; PD‐1, programmed death 1; PD‐L1, programmed death ligand 1.

### 4.2 Targeting CXCL12/CXCR4 to reverse carcinoma‐associated fibroblast immunosuppression

Carcinoma‐associated fibroblasts are an abundant component of the tumor microenvironment and are no longer considered an innocent bystander in cancer progression. Depleting FAP+ carcinoma‐associated fibroblasts in mouse models was shown to be synergistic with vaccine‐based or immune checkpoint‐based immunotherapies in eliciting anti‐tumor immunity and tumor regression.^49^, ^50^ However, therapeutically targeting the stroma of pancreatic cancer is challenging in humans as the cells that comprise this tissue compartment are present throughout the human body and play important roles in normal homeostasis. Thus, pan‐depletion of FAP+ fibroblasts in humans is not a safe or feasible treatment approach.

Feig et al^50^ showed that FAP+ stromal cells produce C‐X‐C motif chemokine ligand 12 (CXCL12), also referred to as stromal‐derived factor‐1. CXCL12 binds to the C‐X‐C chemokine receptor type 4 (CXCR4) and functions to promote cellular chemotaxis.^53^ In pancreatic cancer, CXCL12 is abundant in the tumor microenvironment, whereas CXCR4 has been shown to be expressed on cancer and endothelial cells.^54^ CXCL12 was shown to promote chemotaxis of pancreatic cancer cells expressing CXCR4 in vitro which could be inhibited with AMD3100, an antagonist anti‐CXCR4 mAb, suggesting a mechanism of tumor‐stromal cross‐talk.^55^ CXCR4 is also expressed on hematopoietic cells including macrophages and T cells, and CXCL12 acts as a chemoattractant for these immune cells.^56^

To evaluate whether the interaction of CXCL12 and CXCR4 is significant in the mechanism of FAP+ stromal‐mediated immunosuppression, tumor‐bearing mice were treated with AMD3100, resulting in a T‐cell mediated reduction in tumor growth. When AMD3100 was combined with immune checkpoint blockade with anti‐PD‐L1 therapy, tumor regression occurred with combination treatment but not with checkpoint blockade alone. Mice treated with the combination therapy had increased numbers of intratumoral T cells, suggesting a mechanism behind this treatment effect is improved T‐cell trafficking into the tumor microenvironment.^50^

AMD3100, commercially named Mozobil (Plerixafor injection; Sanofi Oncology, Bridgewater, NJ, USA), is in phase I clinical trial to evaluate its effect on T‐cell infiltration in the tumor microenvironment of patients with advanced pancreatic cancer (Table 1).

### 4.3 Immune checkpoint blockade with agonist CD40 mAb to induce T‐cell immunity

CD40 is a cell surface molecule expressed by immune cells and plays a role in both cellular and humoral immunity. The binding of CD40 to the CD40 ligand expressed on CD4^+^ helper T cells results in activation of antigen‐presenting cells.^57^ Furthermore, triggering CD40 has been shown to enhance the efficacy of vaccines in promoting anti‐tumor immunity.^57^ These findings led to the hypothesis that anti‐CD40 agonist mAb therapy could be used in conjunction with chemotherapy as a novel treatment for pancreatic cancer.^58^

In preclinical studies, CD40 agonist therapy has elicited tumor regression when used in combination with chemotherapy.^59^, ^60^, ^61^ In the KPC model of pancreatic adenocarcinoma, combination treatment with gemcitabine and an agonist CD40 antibody resulted in regression of spontaneous tumors in 30% of mice.^59^ Agonist CD40 treatment in combination with gemcitabine and nab‐paclitaxel also resulted in tumor regression of subcutaneously implanted KPC tumor cells and prolonged overall survival compared to anti‐CD40 therapy or chemotherapy alone.^59^, ^61^

In a phase I clinical trial, CD40 agonist mAb therapy in combination with gemcitabine resulted in tumor response in 19% of patients with unresectable chemotherapy naïve pancreatic cancer.^62^ Tumor biopsies from these patients showed a dearth of TIL and an abundance of tumor infiltrating macrophages.^62^ A second phase I study is aimed at studying the combination of anti‐CD40 therapy with nab‐paclitaxel and gemcitabine in pancreatic cancer and is currently enrolling (Table 1).

In mice with subcutaneous KPC tumors, treatment with anti‐PD‐1 or anti‐CTLA‐4 enhanced the anti‐tumor effects of chemotherapy and agonist CD40 treatment. Thirty‐nine percent of mice treated with anti‐PD‐1, anti‐CTLA4 and an agonist CD40 antibody had long‐term complete tumor remission and prolonged survival. This remarkable tumor response was shown to be T‐cell dependent. CD40 and chemotherapy treatment resulted in changes in the immune microenvironment with a reduction in Treg and an increase in CD8^+^ T cells which was further augmented with immune checkpoint blockade. Importantly, when mice that had a complete response were challenged with tumor cells in the opposite flank, the majority of mice rejected this challenge without additional therapy suggesting developed lasting anti‐tumor immune memory. KPC mice with spontaneous pancreatic tumors who received the combination treatment also had an improved median overall survival compared to control‐treated mice, anti‐PD‐1‐alone treated mice, or CD40/chemotherapy‐treated mice.^7^ Currently, R07009789 (a CD40 agonist mAb) is in phase I clinical trial in conjunction with gemcitabine and nab*‐*paclitaxel for patients with resectable pancreatic cancer (Table 1).

## 5 CONCLUSIONS

1. Patients with pancreatic cancer show poor response to checkpoint blockade with anti‐CTLA‐4 and anti‐PD1/anti‐PD‐L1 immunotherapies.
2. Pancreatic cancer has an abundant desmoplastic stroma which is composed of fibroblasts, immune cells, endothelial cells, and extracellular matrix proteins.
3. The tumor microenvironment is dominated by immunosuppressive cell types (tumor‐associated macrophages, MDSC, and Treg cells) and lacks effector T cells.
4. Carcinoma‐associated fibroblasts have important immunosuppressive effects. Fibroblast activation protein‐α‐positive cells have been shown to be an important mediator in promoting an immunosuppressive microenvironment.
5. Combination treatment strategies that act to stimulate the immune response and break down the barriers of the tumor microenvironment in conjunction with immunotherapy hold promise for improving care for pancreatic cancer patients.

## DISCLOSURE

Conflicts of interest: R.J. Torphy and Y. Zhu declare no conflicts of interest for this article. R.D. Schulick declares patents licensed to Aduro Biotech and GlaxoSmithKline.
